# Evaluation of Serum Homocysteine and Leptin Levels in Patients with Uveitis

**DOI:** 10.4274/tjo.26539

**Published:** 2015-08-05

**Authors:** Arif Emre Elbay, Ayşen Topalkara, Ahmet Elbay, Haydar Erdoğan, Ayşe Vural, Abdi Bahadır Çetin

**Affiliations:** 1 Gazi State Hospital, Clinic of Ophthalmology, Samsun, Turkey; 2 Cumhuriyet University Faculty of Medicine, Department of Ophthalmology, Sivas, Turkey; 3 Pendik State Hospital, Clinic of Ophthalmology, İstanbul, Turkey; 4 Sivas Numune Hospital, Clinic of Ophthalmology, Sivas, Turkey

**Keywords:** Uveitis, Behçet’s, Homocysteine, leptin

## Abstract

**Objectives:**

To evaluate the serum homocysteine (Hcy) and leptin levels in patients with uveitis.

**Ma­te­ri­als and Met­hods:**

The 70 cases included in the study comprised 3 groups: patients with Behçet’s uveitis (BU), patients with non-Behçet’s uveitis (NBU) and healthy controls. Body mass index was calculated for each subject. Serum Hcy and leptin levels were measured. Furthermore, acute-phase reactants including erythrocyte sedimentation rate (ESR), C-reactive protein and neutrophil count were measured.

**Re­sults:**

Serum Hcy levels were 15.04±4.59 µmol/L in the BU group, 15.4±6.87 µmol/L in the NBU group and 13.64±4.72 µmol/L in the control group (p>0.05). The serum leptin levels of male patients in the BU group, NBU group and control group were 4.76±3.54 ng/ml, 6.33±3.74 ng/ml and 5.47±6.33 ng/ml, respectively (p>0.05). When we compared serum leptin levels in female patients and controls, the mean serum leptin concentrations were significantly higher in female BU and NBU patients (24.83±17.62 ng/ml and 28.46±13.90 ng/ml, respectively) than in healthy control volunteers (9.62±6.36 ng/ml, p<0.05). In addition, the ESR value differences between groups were statistically significant (p<0.05).

**Conclusion:**

A larger case series is necessary to investigate serum Hcy and leptin concentrations in uveitis patients.

## INTRODUCTION

Uveitis, an inflammatory ocular disease that can lead to total loss of vision, develops due to autoimmune diseases, infections, toxin exposure and other yet unknown factors.^1,2^ Behçet’s disease (BD) is one of the diseases in which uveitis occurs. BD is a chronic inflammatory disease which can affect all vasculature and perivascular tissues.^3^,^4^ In addition to ocular manifestations, there may be gastrointestinal system, central nervous system and hepatic manifestations, mucocutaneous lesions, and joint involvement.^5,6^

Many factors have been implicated in the etiology of BD, including immune system defects such as increased response from T and B cells to heat shock proteins; changes in neutrophil activation and cytokine levels; endothelial cell dysfunctions; microbial factors; and genetic predisposition.^5,7,8^

Our understanding of the mechanisms of BD pathogenesis and activation continues to expand, and new inflammatory molecules are still being discovered. It has been shown that serum levels of neutrophil-mediated proinflammatory cytokines tumor necrosis factor (TNF), interleukin 1 beta (IL-1β) and IL-8 are elevated during acute exacerbations.^9^ Furthermore, BD patients have exhibited elevated serum levels of homocysteine (Hcy),10 which has been proposed to play an important role in uveal inflammation by increasing nitric oxide (NO) synthesis in endothelial cells. BD patients with and without ocular involvement have shown elevated serum levels of leptin, a peptide hormone produced by the obesity gene that regulates body weight and is structurally and functionally similar to cytokines.^11,12,13^

There are reports of elevated leptin levels in various bodily fluids of patients with non-Behçet’s uveitis (NBU), but our search of the literature did not yield any reports investigating Hcy levels in these patients.^14,15,16^

The aim of this study was to investigate the relationship between serum Hcy and leptin levels in Behçet’s uveitis (BU) and NBU.

## MATERIALS AND METHODS

Forty-five patients who presented to our clinic for the first time or had follow-up examinations in the uveitis unit between May and September 2007 were separated into the BU group and the NBU group. The control group included 25 healthy individuals with no systemic or ocular diseases. BU was diagnosed according to the International Study Group for Behçet’s Disease criteria17 and NBU was diagnosed according to the International Uveitis Study Group criteria.^18^

Uveitis patients were asked to provide medical history including disease duration, medications used and any other existing systemic diseases. Patients’ visual acuity was measured using the Snellen chart and ocular findings were recorded. Patients with a history of systemic hypertension, diabetes mellitus, ischemic heart disease, liver or kidney failure and eating disorders were excluded from the study. Patients whose uveitis had infectious etiology and patients with any other systemic infections were also excluded from the study.

Height, weight and body mass index (BMI) were recorded for patients accepted into the study. BMI was calculated as weight (kg) divided by the square of height (m2). Serum Hcy and leptin levels, hemogram variables erythrocyte sedimentation rate (ESR) and C-reactive protein (CRP) levels were measured. Intergroup comparisons of the data were performed. As leptin levels can show large differences between male and female subjects, data related to leptin were evaluated separately for men and women.

In order to avoid interference with the test results, patients were asked to avoid strenuous exercise, abstain from using cigarettes and alcohol, and fast after their evening meal on the day before blood collection. Any systemic or topical medication use was stopped two weeks before blood collection. Each patient gave a 10 cc sample of venous blood in the morning on an empty stomach, after having rested at least 30 minutes. From the 10 cc of blood, 2.5 cc was used to perform hemogram, ESR and CRP analysis on the same day of collection. The remaining 7.5 cc was stored in a serum separator tube with gel for approximately 1 hour, after which the cellular components were separated by centrifugation for 10 minutes at 3.500 rpm and the serum was stored in Eppendorf tubes at -20 °C until analysis.

Hcy and leptin levels were analyzed by enzyme-linked immunosorbent assay (ELISA) technique. For Hcy, the Axis Homocysteine EIA kit (Axis-Shield Diagnostics Ltd., United Kingdom) was used; for leptin, the DRG Leptin (Sandwich) ELISA (DRG Instruments GmBH, Germany) kit was used. Measurements were conducted according to the manufacturer’s instructions that accompanied the kits, with no changes in technique. Optic absorbance at 450 nm was measured with an automated microplate reader.

The study was approved by the ethics committee. Patients included in the study were informed about the study and provided written informed consent.

Data were analyzed with SPSS version 15.0. Kruskal-Wallis test, Mann-Whitney U test and the chi-square test were used in the statistical analysis. Data in tables are expressed as mean ± standard deviation, number of individuals and percentage. Level of significance was accepted as a=0.05.

## RESULTS

Of the 70 patients included in this study, 23 (17 male, 6 female) had BU and 22 (9 male, 13 female) had NBU. The control group comprised 25 healthy individuals (17 male, 8 female) with no systemic or ocular diseases.

The mean age was 35.52±8.54 (range, 19-50) years in the BU group, 36.90±11.04 (range, 23-65) years in the NBU group, and 31.56±5.00 (range, 20-44) years in the control group. Differences in age and gender distribution between groups were insignificant (p>0.05, Table 1).

Mean serum Hcy levels were 15.04±4.59 µmol/L in the BU group, 15.4±6.87 µmol/L in the NBU group, and 13.64±4.72 µmol/L in the control group. The differences in Hcy level between groups were found to be statistically insignificant (p=0.598). Furthermore, intergroup differences in BMI, neutrophil numbers and CRP values were also insignificant (p>0.05). There were statistically significant differences in ESR between the BU, NBU and control groups (BU: 11.17±13.25 mm/h, NBU: 16.59±21.32 mm/h, control: 6.28±6.56 mm/h; p=0.031). In paired comparison, both the BU and NBU groups showed a statistically significant increase in ESR compared to the control group (p<0.05), while the difference between the BU and NBU groups was insignificant (p>0.05, Table 2).

Differences in average serum leptin levels in male BU and NBU patients compared with males in the control group were statistically insignificant (BU: 4.76±3.54 ng/ml, NBU: 6.33±3.74 ng/ml, control: 5.47±6.33 ng/ml; p>0.05). Intergroup differences in female leptin values were significant (BU: 24.83±17.62 ng/ml, NBU: 28.46±13.90 ng/ml, control: 9.62±6.36 ng/ml; p<0.05). In paired comparison, there were significant differences between the BU and control groups and the NBU and control groups (p<0.05), though the difference between the BU and NBU groups was found to be insignificant (p>0.05, Table 3).

When measured parameters were compared between male and female patients, gender differences in leptin and ESR were statistically significant in both the BU and NBU groups (p<0.05), while the differences in other parameters were insignificant (p>0.05, Tables 4 and 5).

Between males and females in the control group, there were statistically significant gender differences in leptin and BMI were statistically significant (p<0.05); differences in the other parameters were insignificant (p>0.05, Table 6).

## DISCUSSION

This study aimed to evaluate serum levels of Hcy and leptin, which have been proposed to contribute to the endothelial dysfunction and uveal inflammation that occur in both BU and NBU patients. We were unable to find any studies in the literature evaluating the roles of both Hcy and leptin in the development of inflammation. Furthermore, we could not find any reports evaluating serum Hcy levels in NBU patients. Our study has the distinction of being the first to research these issues.

Hcy is a sulfur-containing amino acid that appears during methionine metabolism as methionine is converted to cysteine. Moderate to severe rises in Hcy in the blood or serum may lead to cytokine cascade activation, lipid peroxidation, vascular endothelial damage, prothrombotic surface formation, atherothrombogenesis, thromboembolism and subsequent occlusive vascular disease.^19,20^ NO, an endothelium-derived relaxing factor, is a free oxygen radical produced by endothelial cells as a response to immunologic, infectious and inflammatory stimuli such as cytokines, interferon gamma (IFN-γ), lipopolysaccharides and endotoxin.^21,22^ Increased NO production resulting from the activation of cytokines by Hcy may be pathophysiologically related to BD and uveitis.^10,23,24^

Considering this information, many studies have been conducted regarding serum Hcy levels in BD patients. While most investigators reported significant increases in the serum Hcy levels of BD patients compared to healthy individuals,^10,11,12,13,14,15,16,17,18,19,20,21,22,23,24,25,26,27,28,29,30 ^ some did not find a relationship between BD and serum Hcy levels.^31,32,33,34^

Among the smaller number of studies regarding ocular involvement in BD, significantly elevated serum Hcy levels in ocular BD patients were reported in five studies;^10,27,28,35,36^ a decrease in Hcy levels and uveitis attacks was observed following folate treatment in one study;^37^ and in another study no relationship between Hcy and ocular involvement in BD was found.^38^

In a study by Er et al.10 including 27 BD patients with ocular involvement, 16 BD patients without ocular involvement and 25 healthy controls, mean plasma Hcy levels were measured as 18.25±4.20 ng/ml, 13.53±3.34 ng/ml and 7.96±2.96 ng/ml, respectively, and the differences were found to be statistically significant for both patient groups when compared with controls (p<0.01 and p<0.001, respectively). Okka et al.^27^ found higher serum Hcy levels in patients with BU compared to patients with inactive BD and healthy controls. Durmazlar et al.^28^ compared Hcy levels in 18 BD patients with ocular involvement and 52 BD patients without ocular involvement and reported higher Hcy levels in BD patients with ocular involvement than in those without (median values: 21.59 ng/ml [range, 14-39.3 ng/ml] and 14.15 ng/ml [range, 6.4-38 ng/ml], respectively, p<0.001).

In contrast to the data found in the literature, although the BU patients in our study had higher serum Hcy levels than the control group, the difference was not statistically significant.

Another agent suspected in the pathogenesis of BD and BU is leptin, a protein encoded by the ob gene. Leptin has been reported to regulate energy metabolism, increase vascular permeability, induce angiogenesis, and regulate wound healing, and is proposed to possibly play an important role in inflammation.^39^ In animal experiments as well as studies with human volunteers, elevated serum leptin levels were observed following the administration of cytokines.^40,41,42^

It has been shown that there are leptin receptors on the surface of endothelial cells, and that leptin increases NO release from these cells. Cytokines stimulate leptin production during inflammation; leptin then causes endothelial cells to release NO. Therefore, it is believed that some of the biological activities of NO are regulated by leptin.^13^

In contrast, there are also reports that leptin has protective anti-inflammatory properties. In one study, leptin was found to have an anti-inflammatory effect in experimental colitis; in another study it was shown that leptin replacement restored impaired endothelial cell function.^43,44^

Taking these characteristics of leptin into account, several studies were conducted investigating serum leptin levels in BD patients.^11,13,45,46^ While Kavuncu et al.^46^ found no statistically significant difference in serum leptin levels between BD patients and healthy controls, in the other three studies it was reported that serum leptin levels of BD patients were elevated and that leptin may play a role in the pathogenesis of BD via endothelial function.^11,13,45^

As serum leptin levels are higher in women and increase with body fat proportion, we evaluated leptin and related measurements separately for men and women in this study. Serum leptin levels were found to be markedly higher in women than men in all three study groups. Although no significant difference was found between the serum leptin levels of male BU patients and controls, female BU patients showed significantly elevated serum leptin levels. Our inability to detect differences in male patients’ serum leptin concentrations may have been due to the narrow range of their’ leptin levels and the techniques we used.

Our search of the literature yielded no studies evaluating serum Hcy levels in NBU patients, though we found four studies investigating the association between NBU and leptin.^14,15,16,47^ In one of these studies including 30 ankylosing spondylitis patients, no significant differences were found between the serum leptin levels of patients with and without a history of uveitis.^47^ In another study comparing patients with active Vogt-Kayanagi-Harada syndrome, patients with the inactive disease and healthy individuals, elevated serum leptin levels were found in patients with active disease.^15^ Kalariya et al.^14^ reported increases in leptin and other cytokines in the aqueous humour of rats triggered by endotoxin. Kükner et al.^16^ administered intravitreal bovine serum albumin injections to pigs and found increased leptin expression in the pigs’ retina, choroid, sclera and episclera.

In our study, similar results were obtained when the serum Hcy and leptin levels of the NBU and BU groups were compared with those of the control group. When comparing the NBU and BU groups, the NBU patients had higher levels of both Hcy and leptin, though the difference was not statistically significant. The fact that the NBU patients were not a homogenous group is one limitation of our study.

Some studies report comparisons of patients with active and inactive disease, or comparisons of patients’ data in active and inactive disease periods.^10,11,13,14,26,27,28,31^ Furthermore, increased leptin level has been observed with acute inflammatory stimuli like major surgery, though a leptin peak was not observed in chronic conditions such as rheumatoid arthritis and inflammatory bowel disease.^48,49,50^

The lack of distinction between active/inactive or acute/chronic disease in our patients is another limitation of our study, and may explain why we were unable to detect significant increases in serum Hcy and leptin levels in male patients.

Parameters such as ESR, neutrophil count, and serum levels of CRP, endotelin-1, NO, TNF-α, α-1 antitrypsin, and α-2 macroglobulin, which increase in active inflammation and some inflammatory diseases, have been found at elevated levels in uveitis patients.^10,11,13,27,28,46^ In addition, most of the studies revealed a correlation between these parameters and Hcy or leptin levels.^10,11,13,27,28^

One of the parameters evaluated in our study was BMI, and there are studies reporting no significant difference in BMI between BD and control groups.^13,46^ However, a correlation between BMI and leptin in Behçet’s patients with and without ocular involvement has been reported.^11^ In our study no significant intergroup differences in BMI were found, and leptin levels were independent of BMI.

While we also found no significant differences between groups in terms of neutrophil count and CRP, an increase in ESR was observed in the BU and NBU groups compared to the control group. Our inability to detect significant increases in CRP and neutrophil count may be attributable to the fact that patients with active disease status were not evaluated separately.

To further evaluate Hcy and leptin levels in uveitis patients, a larger case series is needed.

## Figures and Tables

**Table 1:**
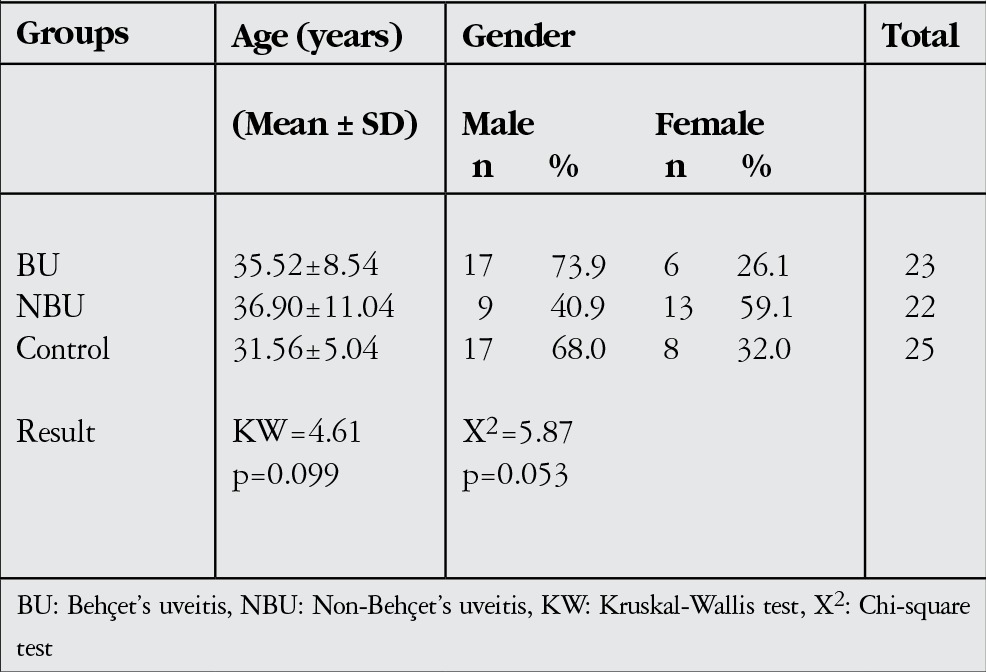
Age and gender distribution of the study population

**Table 2:**
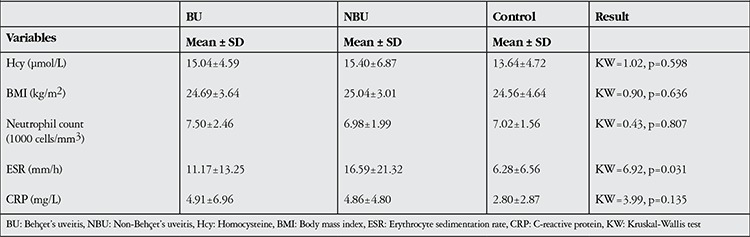
Serum homocysteine levels, body mass index, neutrophil count, sedimentation and C-reactive protein values and distributions

**Table 3:**
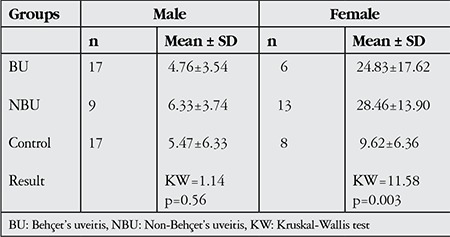
Serum leptin level (ng/ml) values and distribution

**Table 4:**
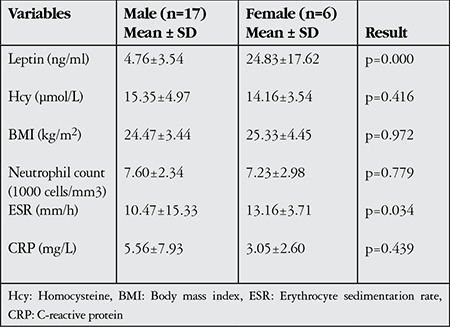
Parameter distributions of the male and female patients in the Behçet’s uveitis group

**Table 5:**
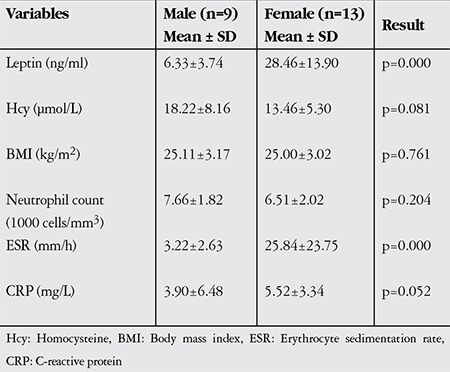
Parameter distributions of the male and female patients in the Non-Behçet’s uveitis group

**Table 6:**
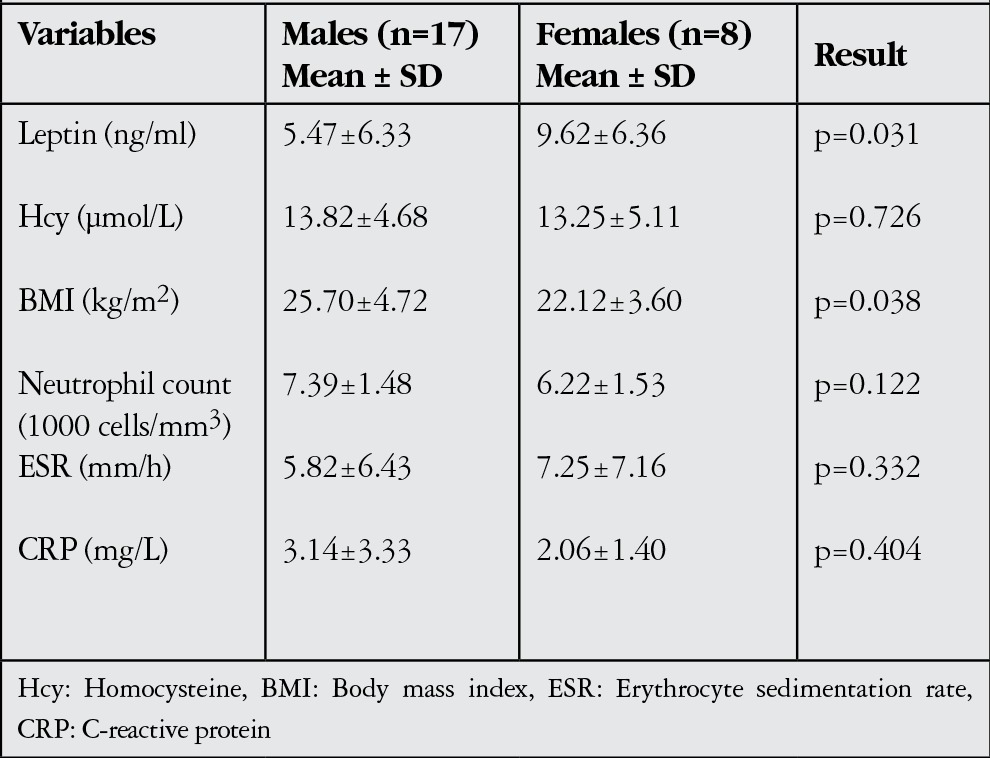
Parameter distributions of the male and female individuals in the control group
